# Germline variants in cancer genes in high-risk non-*BRCA* patients from Puerto Rico

**DOI:** 10.1038/s41598-019-54170-6

**Published:** 2019-11-28

**Authors:** Julie Dutil, Jamie K. Teer, Volha Golubeva, Sean Yoder, Wei Lue Tong, Nelly Arroyo, Rachid Karam, Miguel Echenique, Jaime L. Matta, Alvaro N. Monteiro

**Affiliations:** 1 Cancer Biology Division, Ponce Research Institute, Ponce Health Sciences University, Ponce, PR USA; 2 Department of Biostatistics and Bioinformatics, H. Lee Moffitt Cancer Center and Research Institute, Tampa, FL USA; 3 Cancer Epidemiology Program, H. Lee Moffitt Cancer Center and Research Institute, Tampa, FL USA; 4 Molecular Genomics Core, H. Lee Moffitt Cancer Center and Research Institute, Tampa, FL USA; 5 University of South Florida Morsani College of Medicine, Tampa, FL USA; 6 Ambry Genetics, Aliso Viejo, CA USA; 7 Auxilio Cancer Center, Auxilio Mutuo Hospital, San Juan, PR USA

**Keywords:** Cancer genetics, Genetics research

## Abstract

Inherited pathogenic variants in genes that confer moderate to high risk of breast cancer may explain up to 50% of familial breast cancer. This study aimed at identifying inherited pathogenic variants in breast cancer cases from Puerto Rico that were not linked to *BRCA1* or *BRCA*2. Forty-eight breast cancer patients that met the clinical criteria for *BRCA* testing but had received a negative *BRCA1*/2 result were recruited. Fifty-three genes previously implicated in hereditary cancer predisposition were captured using the BROCA Agilent cancer risk panel followed by massively parallel sequencing. Missense variants of uncertain clinical significance in *CHEK2* were evaluated using an *in vitro* kinase assays to determine their impact on function. Pathogenic variants were identified in *CHEK2*, *MUTYH*, and *RAD51B* in four breast cancer patients, which represented 8.3% of the cohort. We identified three rare missense variants of uncertain significance in *CHEK*2 and two variants (p.Pro484Leu and p.Glu239Lys) showed markedly decreased kinase activity *in vitro* comparable to a known pathogenic variant. Interestingly, the local ancestry at the *RAD51B* locus in the carrier of p.Arg47* was predicted to be of African origin. In this cohort, 12.5% of the *BRCA*-negative breast cancer patients were found to carry a known pathogenic variant or a variant affecting protein activity. This study reveals an unmet clinical need of genetic testing that could benefit a significant proportion of at-risk Latinas. It also highlights the complexity of Hispanic populations as pathogenic factors may originate from any of the ancestral populations that make up their genetic backgrounds.

## Introduction

Women with a pathogenic variant in *BRCA1* or *BRCA*2 are at substantially higher risk of developing breast, ovarian and other cancers^1^. For these women, risk reduction options include increased surveillance, chemoprevention and prophylactic surgery^2^. While the identification of a *BRCA* pathogenic variant constitutes a clear benefit for probands and their relatives^3,4^, a proportion of at-risk families do not present pathogenic variants in *BRCA1* or *BRCA*2 and their genetic risk of breast cancer remains unexplained.

Significant progress has been made in the identification of inherited genetic factors underlying hereditary cancers. Pathogenic variants in *PTEN*, *TP53*, *CHEK2*, *ATM*, *NBS1*, *RAD50*, *BRIP1* and *PALB2*, amongst others, have also been shown to confer moderate to high risk of breast cancer^5,6^. Recently, several commercial laboratories have replaced *BRCA* testing with extended panels of cancer susceptibility genes^7^. Yet, for many of the genes tested, there are no approved guidelines dictating the optimal prevention or treatment strategy should a loss of function variant be identified^8^.

The Hispanic/Latino population is the fastest growing ethnic minority in the US and is predicted to account for 35% by 2050^9^. Although breast cancer (BC) incidence and mortality are lower compared to most ethnic groups in the US, the outcome and prognosis in Hispanic women are worse^10^. The prevalence and spectrum of *BRCA* variants varies significantly within Latin America and the Caribbean, with little overlap in the pathogenic variants most frequently reported in each country^11^. In Puerto Rico, our previous work estimated that a majority of the *BRCA* cases are explained by few recurrent founder mutations^12,13^. This study aimed at identifying hereditary cancer variants in *BRCA*-negative breast cancer cases from Puerto Rico using the BROCA panel of cancer predisposition genes^14^.

## Methods

### Study participants

The study protocol was approved by the Institutional Review Board of Ponce Research Institute (#120829-JD) and Moffitt Cancer Center (#MCC17404) and all methods were performed in accordance with relevant guidelines and regulations. Participants were recruited between September 2012 and September 2014 from a private surgery practice located in San Juan, Puerto Rico. Evaluation of the potential study participants was conducted by a single surgeon. All women were diagnosed with breast cancer and met the NCCN (National Comprehensive Cancer Network current at the time of the study design, version 1.2012) guidelines for *BRCA* testing, but had received a negative *BRCA* clinical test result. All study participants provided informed consent and received an orientation about genetics and hereditary cancers. Trained study personnel gathered a three-generation pedigree depicting family history of cancer. Participants whose grandparents had not been born in Puerto Rico were excluded. A complete surgery clinical pathology report was obtained for the tumor(s) of each participant. Not all tumors were evaluated by the same pathologist. A total of 97 women were interviewed, 56 (57.7%) met the inclusion criteria and agreed to participate. In addition, a sample containing a known *BRCA2* c.3922G > T (p.Glu1308Ter) pathogenic variant was included as a blinded control. A cohort of 500 women from Puerto Rico that do not have a cancer diagnosis and that received a negative breast cancer screening mammography within 6 months of enrollment was used to assess frequency of variants of interests in the population of Puerto Rico.

### BRCA1/2 large rearrangement screening

To ensure that all women included were true negative for *BRCA1/2* pathogenic variants, comprehensive screening for large rearrangements was performed by multiplex ligation-dependent probe amplification (MLPA). The MLPA kits P002-D1 (*BRCA1* NM_007294.3) and P045-B3 (*BRCA2* NM_000059.3) and the protocol for the final assessment of allele dosage were used as described by the manufacturer (MRC-Holland, Amsterdam, the Netherlands)^15^.

### Sequencing

Genomic DNA was extracted using Paxgene blood DNA Kit (Qiagen, Valencia, CA) and used for the preparation of paired-end libraries with 200 base pairs (bp) inserts. An oligonucleotide pool targeting exons, non-repeating introns and selected promoter regions of the genes of interest was used for enrichment (SureSelect). Following capture, samples were sequenced on an Illumina MiSeq. The total genomic region covered was 1.1 million bp (Mbp). The 53 genes of the BROCA (http://tests.labmed.washington.edu/BROCA_versions) panel are listed in Supplementary Fig. 1. This panel was developed for the evaluation of patients at risk of hereditary cancers, it is not restricted to BC susceptibility genes. Forty-eight samples achieved proper QA/QC standards and remained for further analyses.

### Alignment, variation detection, annotation

Sequence reads were aligned to the reference human genome (hs37d5) with the Burrows-Wheeler Aligner (BWA)^16^, and duplicate identification, insertion/deletion realignment, quality score recalibration, and variant calling were performed with PICARD (http://picard.sourceforge.net/), the Genome Analysis ToolKit (GATK)^17^. Sequence variants were annotated using ANNOVAR^18^. Additional contextual information was incorporated, including allele frequency in other studies such as 1000 Genomes (Phase 3 20130502, downloaded 11/2015), NHLBI Exome Sequence Project (ESP6500 v2, downloaded 8/2013), and the Exome Aggregation Consortium (release 0.2, downloaded 1/2015). *In silico* functional impact predictions, and observed impacts were assessed from databases like ClinVar (http://www.ncbi.nlm.nih.gov/clinvar/) (release 9/2016), the Catalog Of Somatic Mutations In Cancer (COSMIC) (v68), RegulomeDB^19^, and FunSeq^20^. Analyses were performed using custom bash, Perl, R scripts, and VarSifter^21^. Variants distribution plots were realized using Lollipops (https://zenodo.org/badge/latestdoi/20224/pbnjay/lollipops). We obtained an average of 2.7 million reads per sample, 99% aligning to the human genome and an average depth of coverage of 83.9 (Supplementary Fig. 1). After filtering for genotype quality, read depth and 1 kG population frequencies, we identified a total of 2,270 single nucleotide variants and 490 indels with allelic frequency of less than 1% in the 1 kG Admixed American reference populations (Supplementary Fig. 2).

### Variant classification

Each non-synonymous coding variant was assessed individually for classification into the following categories: (1) likely benign or benign, (2) likely pathogenic or pathogenic, (3) variant of unknown significance (VUS). This classification did not distinguish variants of high penetrance from those of moderate penetrance sometimes refered to as ‘risk factor’ in public databases. First, 16 variants that have been classified as benign or likely benign by expert panels (ENIGMA for *BRCA1* and *BRCA2*^22^ and Insight for Lynch Syndrome genes^23^) were not further investigated (n = 17). For the remaining variants, those that had a frequency >0.5% in any of the gnomAD^24^ populations (African, East Asian, non-Finnish European, Latino, South Asian) were filtered from the analysis unless they had been reported as likely pathogenic or pathogenic on ClinVar^25^.Finally, the remaining variants were classified based on the ACMG criteria^26^. The frequency of likely pathogenic/pathogenic variants and VUS identified in NCCN clinically actionable gene was assessed in a cohort of 500 women that do not have cancer history by multiplex PCR using a Sequenom analyzer. *In Silico* predictions of functional significance was obtained for each non-synonymous variant using the following algorithms: fathmm^27^, MutationAssessor^28^, PolyPhen-2^29^ and SIFT^30^.

### Ancestry

Global ancestry proportions were estimated using a panel of 106 ancestry informative SNPs that can discriminate indigenous American, African, and European ancestry^31^. Genotyping was done by multiplex PCR using a Sequenom analyzer as previously described^31^. For each individual, respective proportions of European, African and Native American ancestry were estimated using ADMIXTURE^32^. In pathogenic variant carriers, local ancestry at the site of the genes of interest was extracted from Affymetrix Axiom UK array genotypes that had been phased using Shape-it^33^ and for which locus-specific ancestry was determined using RFMix^34^.

### CHK2 kinase assays

To determine the impact of *CHEK2* missense variants we inserted p.Glu239Lys, p.Pro484Leu, and pArg519Leu variants in a wild type human *CHEK2* sequence in pCMV2-FLAG by site directed mutagenesis using QuikChange II kit (Agilent, Santa Clara, CA) (primers available upon request). We also constructed two known pathogenic variants, p.His143Tyr and p.Ile157Thr, to serve as negative controls. Constructs were confirmed by Sanger sequencing.

Human embryonic kidney 293FT cells were transfected with empty pCMV2-FLAG vectors, or with expression vectors containing wild-type CHK2 or variants using Fugene HD (Promega, Madison, WI). After ~48 h cells were exposed to 6 Gy of ionizing radiation (IR) and lysates were collected after 1 h in RIPA buffer (150 mM NaCl, 10 mM Tris-Cl [pH 7.4], 5 mM EDTA, 0.1% sodium dodecyl sulfate, 1% Triton X-100, 0.1% sodium deoxycholate). Levels of total and phosphorylated CHK2 in lysates were analyzed by immunoblotting on a 10% SDS-PAGE gel. The following antibodies were used: anti-phospho-Serine 516 CHK2 (Cell signaling), anti-phospho-Threonine 68 CHK2 (Cell signaling), anti-CHK2 (EMD Millipore) and anti-β-actin (Santa Cruz Biotechnology).

For immunoprecipitation and kinase assays cell lysates were incubated at 4 °C for 2 h with anti-FLAG M2 affinity gel. Immunoprecipitates were washed three times with RIPA buffer and used for immunoblotting with anti-phospho-CHK2 and anti-CHK2. For kinase assay, the beads were incubated with Kinase Buffer (20 mM HEPES, pH 7.4, 10 mM MgCl_2_, 10 mM MnCl_2_, 1 mM PMSF, 40 μM cold ATP, and 13 μCi [γ-^32^P] ATP) and bacterially expressed GST-CDC25C peptide (aa 200–256; containing the phosphorylation site for CHK2) as an exogenous substrate. Kinase reactions were incubated for 30 min at 30 °C. Samples were resolved on a 12% SDS-PAGE gel.

### Statistical analyses

Descriptive and statistical analyses were conducted in R Studio^35^.

## Results

### Characteristics of the study cohort

The description of demographic, hormonal and clinical characteristics of the study cohort is presented in Supplementary Table 1. Over half of the study participants (56.2%) had their breast cancer diagnosis before the age 50. The majority of the tumors were invasive ductal carcinomas (65.9%), of lower stages; grade I and II represented 77.4% of the invasive tumors, size below 2 cm (76.9%) and luminal A or B subtype (88.2%) (Supplementary Table 1).

### Variants

There were 158 coding variants: 2 truncating, 2 deletions, 106 missense and 48 synonymous variants. Each affected proband had on average 0.8 non-synonymous coding VUS, ranging from 0 to 4.

**Figure 1:**
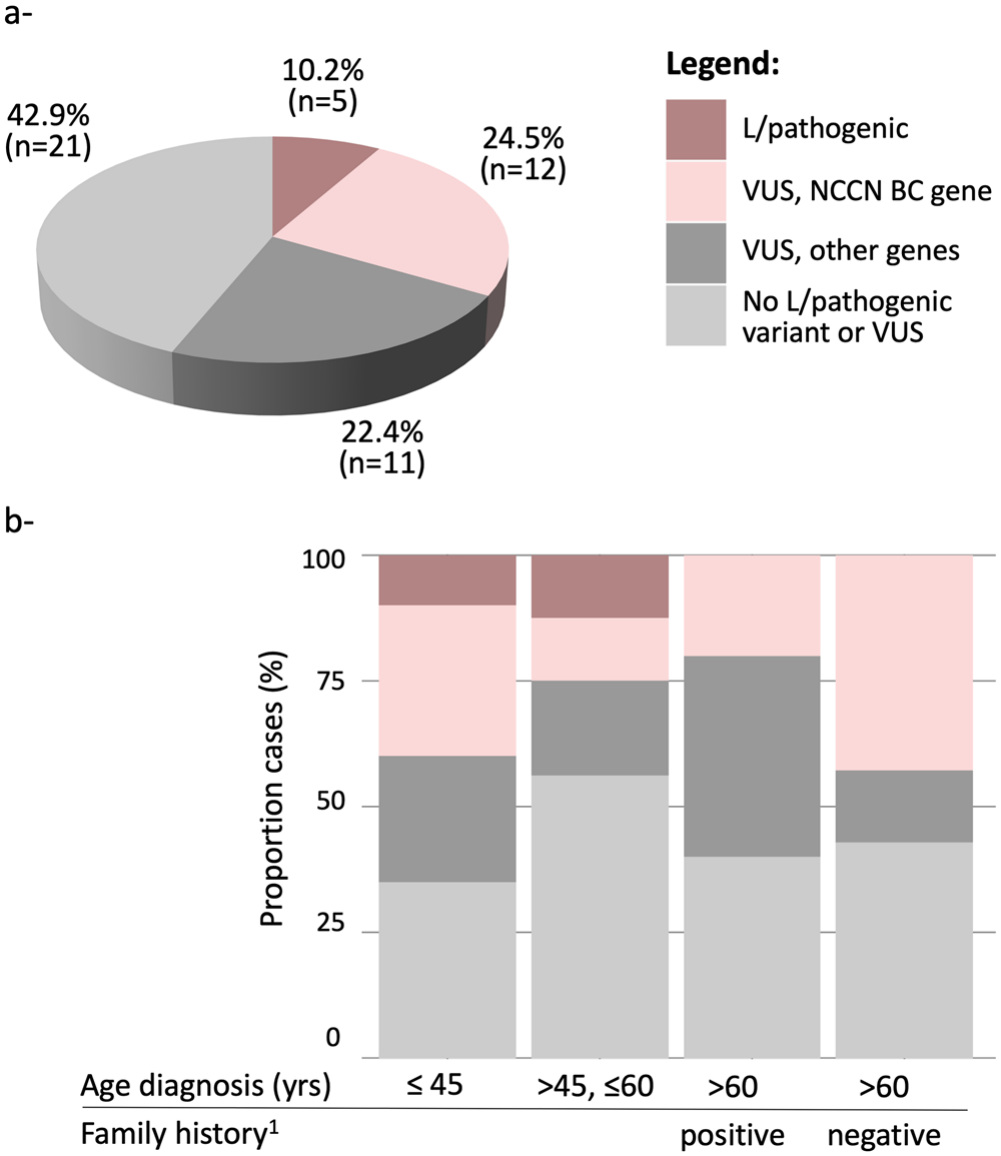
(**a**) Proportion of unrelated BC patients by carrier status and (**b**) distribution after grouping by age of onset and family history characteristics. Carriers categories are: harboring at least one coding variant classified as likely pathogenic or pathogenic (L/Pathogenic), VUS in NCCN clinically actionable genes, VUS in other genes, or no L/Pathogenic variant or VUS identified. Early onset breast cancer refers to individuals for which diagnosis was at 45 years of age or less. Positive family history includes only individuals for which a first, or second degree relative was diagnosed with an early onset breast cancer. NCCN clinically actionable BC genes are *ATM*, *BRCA1*, *BRCA2*, *CDH1*, *CHEK2*, *NBN*, *NF1*, *PALB2*, *PTEN*, *STK11*, and *TP53* (NCCN Guidelines for Genetic/Familial High-Risk assessment v3.2019^25^). The complete list of genes included in the BROCA panel is available in the methods section. Coding variants included truncating, missense, deletions and duplications. Synonymous variants were excluded from this analysis. BC breast cancer; VUS variant of uncertain significance.

The study participants were classified according to their carrier status of a variant affecting the genes coding regions (truncating, nonsense and missense variants): Group I (n = 5; 10.2%), patients harboring at least one coding variant classified as likely pathogenic or pathogenic (L/Pathogenic) in any gene. Group II (n = 12, 24.5%), patients with VUS in breast cancer susceptibility genes classified as “clinically actionable” by NCCN^27^. Group III (n = 11, 22.4%), patients with variants in any other gene in the panel. Group IV (n = 21, 42.9%), patients with no pathogenic variant or VUS found in the coding sequences (Fig. 1).

To assess the prior probability of being carrier, each proband was grouped according to age of onset and/or family history of early onset breast cancer (at or before age 45). There was no significant difference in the distribution of carrier status after grouping probands by age of onset and/or family history of early onset breast cancer (*P* = 0.8, Fig. 1). It is noteworthy however that 3 out of 4 carriers of a L/pathogenic variant had a breast cancer diagnosis at or before 50 years of age.

### Pathogenic variants

**Figure 2:**
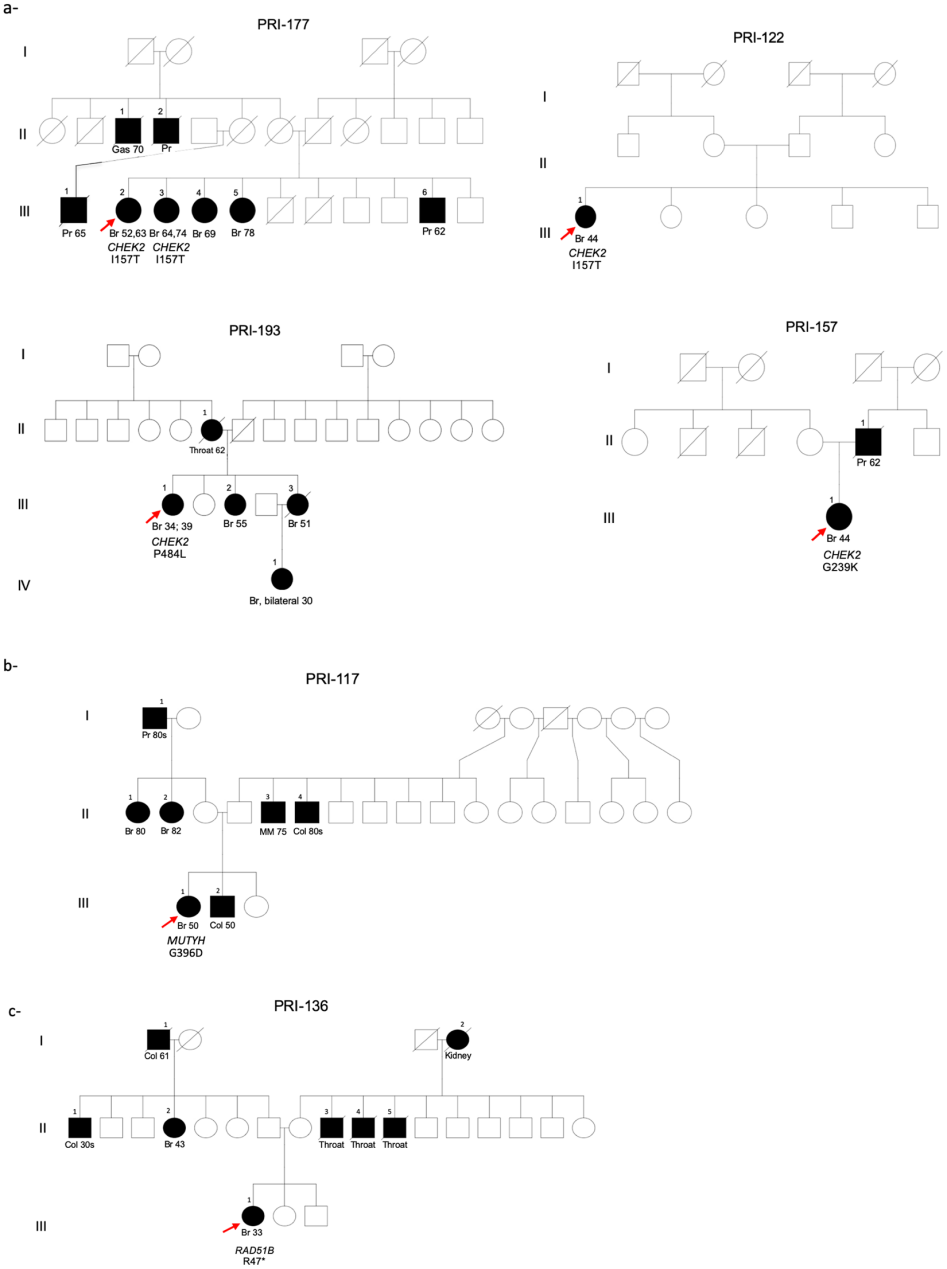
Family history of cancer (site and age of diagnosis, dx) in relatives of carriers of a known or predicted pathogenic variant in *CHEK2* (**a**), *MUTYH* (**b**), and *RAD51B* (**c**). Sites of carcinoma are Br breast cancer, Col colorectal cancer, Gas gastric cancer, MM multiple myeloma, Pr prostate cancer. Probands are designated by a red arrow.

After excluding the *BRCA2* p.Glu1308Ter carrier (blind control), which was properly identified, four probands in 48 (8.3%) were found to carry a pathogenic variant in a non-*BRCA* breast cancer gene (Table 1). *CHEK2* p.Ile157Thr was observed in two unrelated individuals (Fig. 2). Family history for the first patient was characterized by multiple first-degree relatives with late onset breast cancer, prostate and gastric cancer. The other carrier was diagnosed with breast cancer at the age of 44 years with no known family history of cancer.

**Table 1:** Pathogenic and VUS non-synonymous variants in NCCN clinically actionable breast cancer genes.

Gene	Variant (chr:position)	dbSNP ID	HCVS (c)	HCVS (p)	N. obs^a^	ClinVar	ACMG^b^	Highest gnomAD population MAF	Puerto Rico non-cancer controls
*ATM*	11:108098389	rs778201041	c.38G > A	p.Arg13His	1	VUS	VUS	3.3e-05 (South Asian)	0
	11:108121787	rs35963548	c.1595G > A	p.Cys532Tyr	1	L/Benign, VUS	VUS	8.2e-04 (Latino)	0
	11:108160480	rs138327406	c.4388T > G	p.Phe1463Cys	1	VUS	VUS	1.1e-03 (Latino)	2.0e-03
	11:108196144	rs879254132	c.6680G > A	p.Arg2227His	1	L/Benign, VUS	VUS	8.8e-06 (European)	0
	11:108202716	rs199915459	c.7740A > C	p.Arg2580Ser	2	VUS	VUS	2.3e-04 (Latino)	8.8e-03
	11:108225561	rs587782149	c.8810T > C	p.Val2937Ala	1	VUS	VUS	1.2e-03 (Latinos)	0
*BRCA2*	13:32913588	rs80358732	c.5096A > G	p.Asp1699Gly	1	VUS	VUS	7.0e-05 (Latino)	0
	13:32936785	rs80359020	c.7931A > G	p.Asn2644Ser	1	VUS	VUS	8.8e-06 (European)	0
*CDH1*	16:68853185	rs553907248	c.1568A > G	p.Tyr523Cys	2	VUS	VUS	1.1e-04 (Latino)	5.0e-03
*CHEK2*	22:29121087	rs17879961	c.599T > C	p.Ile157Thr	2	L/Pathogenic, VUS	L/Pathogenic	5.3e-03 (European)	0
	22:29107974	rs121908702	c.715G > A	p.Glu239Lys	1	VUS	VUS	2.5e-04 (Latino)	2.0e-03
	22:29090030	rs564605612	c.1451C > T	p.Pro484Leu	1	VUS	VUS	2.6e-04 (Latino)	2.1e-03
	22:29083961	rs587780180	c.1556G > T	p.Arg519Leu	1	VUS	VUS	4.6e-04 (Latino)	NA
*NBN*	8:90982716	rs922057169	c.772G > C	p.Glu258Gln	1	VUS	VUS	NA	NA

^a^Number of observations in unrelated individuals, all variants were present in the heterozygous form. ^b^Classification according to the American College of Medical Genetics and Genomics standards^26^. L/Benign likely benign or benign, L/pathogenic likely pathogenic or pathogenic, MAF Minor allele frequency.

The patient harboring a pathogenic variant in *MUTYH* (p.Gly396Asp, PRI-117 III-1) was diagnosed with breast cancer at the age of 50 and had a brother with early onset colorectal cancer. A truncating variant, p.Arg47* was identified in *RAD51B* predicted to result in a protein lacking ~90% of the coding region. This patient (PRI-136 III-1) was diagnosed with an invasive ductal carcinoma at age 33 and family history included early onset breast and colorectal cancer. Pedigrees representing family histories of patients carrying known pathogenic variants are depicted in Fig. 2.

### Variants of uncertain clinical significance

The location of coding variants in each gene is depicted in Supplementary Fig. 3. Tables 1 and 2 present all the non-synonymous variants classified as L/Pathogenic and VUS by ClinVar^25^ and/or using ACMG^26^ criteria. The highest number of VUS was observed in *ATM* (n = 6) (Table 1), a clinically actionable breast cancer gene. Despite having received a negative *BRCA1*/*BRCA2* test result, two missense VUS were identified in *BRCA2*.

**Table 2:** Pathogenic and VUS non-synonymous variants in other hereditary cancer genes.

Gene	Chr:position	dbSNP ID	HCVS (c)	HCVS (p)	N. obs^a^	ClinVar	ACMG	Highest gnomAD population MAF	1000Genomes PUR N obs. (het.)
*APC*	5:112177928	rs186926737	c.6637A > G	p.Met2213Val	1	VUS	VUS	5.0e-04 (African)	0
*BLM*	15:91304122	rs192491153	c.1519G > A	p.Glu507Lys	1	L/Benign, VUS	VUS	8.7e-05 (Latino)	3
	15:91326057	rs758692622	c.2561G > A	p.Ser854Asn	1	VUS	VUS	8.7e-05 (Latino)	0
	15:91358475	rs557057587	c.4220G > A	p.Arg1407Lys	2	NA	VUS	2.5e-04 (Latino)	1
*BMPR1A*	10:88683138	rs55932635	c.1348G > A	p.Val450Met	1	L/Benign, VUS	VUS	4.3e-04 (East Asian)	0
*BRCC3*	X:154305469	rs28997578	c.220A > G	Ile74Val	1	NA	VUS	1.2e-3 (European)	1
*CHEK1*	11:125503187	rs760379838	c.554A > G	p.His185Arg	1	NA	VUS	2.4e-05 (European)	0
*GALNT12*	9:101589171	rs773546298	c.669C > G	p.Leu227Val	1	VUS	VUS	2.83e-03 (Latino)	0
*GEN1*	2:17954003	rs148607792	c.905G > A	p.Arg302His	1	NA	VUS	4.6e-03 (European)	1
	2:17963043	rs531130654	c.2564G > A	p.Arg855Lys	1	NA	VUS	1.7e-04 (South Asian)	0
*MRE11A*	11:94219093	rs587780140	c.311G > C	p.Ser104Thr	1	VUS	VUS	5.7e-05 (Latino)	0
*MSH6*	2:48027040	rs786203970	c.1921_1923delGAA	p.Glu641del	1	VUS	VUS	NA	NA
*MUTYH*^b^	1:45800158	rs1057517460	c.62C > T	p.Ala21Val	1	VUS	VUS	NA	NA
	1:45797228	rs36053993	c.1187G > A	p.Gly396Asp	1	L/Pathogenic	L/Pathogenic	4.9e-03 (European)	2
*PIK3CA*	3:178916753	rs190372148	c.140A > G	p.His47Arg	2	VUS	VUS	4.5e-04 (African)	3
*PMS2*	7:6027134	rs778482303	c.1262G > A	p.Arg421Gln	1	VUS	VUS	2.8e-05 (Latino)	0
*POLD1*	19:50902657	rs141319800	c.232C > T	p.Arg78Cys	1	VUS	VUS	2.4e-04 (African)	0
*POLE*	12:133245025	rs36120395	c.2090C > G	p.Pro697Arg	2	L/Benign, VUS	VUS	1.2e-03 (European)	0
*PPM1D*	17:58678214	rs148074313	c.439C > G	p.Leu147Val	1	NA	VUS	9.6e-04 (Latino)	1
*PRSS1*	7:142459867	rs762545562	c.443C > T	p.Ala148Val	1	NA	VUS	1.8e-05 (European)	0
*RAD51B*	14:68292235	rs200355697	c.139C > T	p.Arg47*	1	VUS	L/Pathogenic	7.2e-04 (South Asian)	0
	14:68353784	rs28908168	c.619G > T	p.Val207Leu	2	NA	VUS	2.9e-03 (European)	0
*SDHB*	1:17349152	rs201098090	c.716C > G	p.Ser239Cys	1	VUS	VUS	1.3e-04 (South Asian)	0
	1:17355201	rs934514080	c.317A > G	p.Asn106Ser	1	VUS	VUS	1.1e-04 (African)	0

^a^Number of observations in unrelated individuals, all variants were present in the heterozygous form. ^b^Classification according to the American College of Medical Genetics and Genomics standards^26^. ^c^MUTYH-associated polyposis is inherited as an autosomal recessive syndrome. Het. Heterozygous, L/Benign likely benign or benign, L/pathogenic likely pathogenic or pathogenic, MAF Minor allele frequency.

All of the non-synonymous variants identified have previously been reported either in ClinVar^25^ or in the gnomAD^24^ databases. Despite a low observed prevalence in gnomAD, *BLM* p.Arg1407Lys and *CDH1* p.Tyr523Cys were each present in two unrelated individuals in this cohort. Variants *ATM* p.Val2937Ala and *BLM* p.Ser854Asn, have been observed in the gnomAD Latino population exclusively (Supplementary Table 2). For variants that have been reported in more than one gnomAD populations, some exhibited significant differences in frequencies, notably *BLM* p.Arg1407Lys and *CDH1* p.Tyr523Cys, which were 32 and 14 times more frequent in gnomAD Latinos than in non-Finnish European, respectively.

*In Silico* prediction analysis of functional significance led to conflicting interpretation for most of the non-synonymous variants, with the exception of *ATM* p.Arg2227His, *MUTYH* p.Glu396Asp, and *SDHB* p.Ser239Cys which were predicted damaging by all four models tested (Supplementary Table 3).

In 27.1% of the cohort (n = 13), no known pathogenic variant or coding VUS were identified. Among those, PRI-185 stands out for having strong family history of cancer including early onset breast cancer, ovarian cancer and colorectal cancer (*data not shown*). For this individual, the prior risk of being a *BRCA* carrier was estimated at 14.7% using the BRCAPRO model (*data not shown*).

### Functional impact of the CHEK2 variants

In addition to the pathogenic CHK2 variant found in two unrelated probands (Table 1), we identified three rare missense variants of uncertain significance (p.Glu239Lys, p.Pro484Leu, and p.Arg519Leu) (Table 2). To assess their impact on CHK2 stability and activity we expressed epitope-tagged CHK2 constructs containing the variants in 293T HEK cells and compared them to the wild-type CHK2 construct (positive control) and with known CHK2 pathogenic variants p.His143Tyr and p.Ile157Thr.

**Figure 3:**
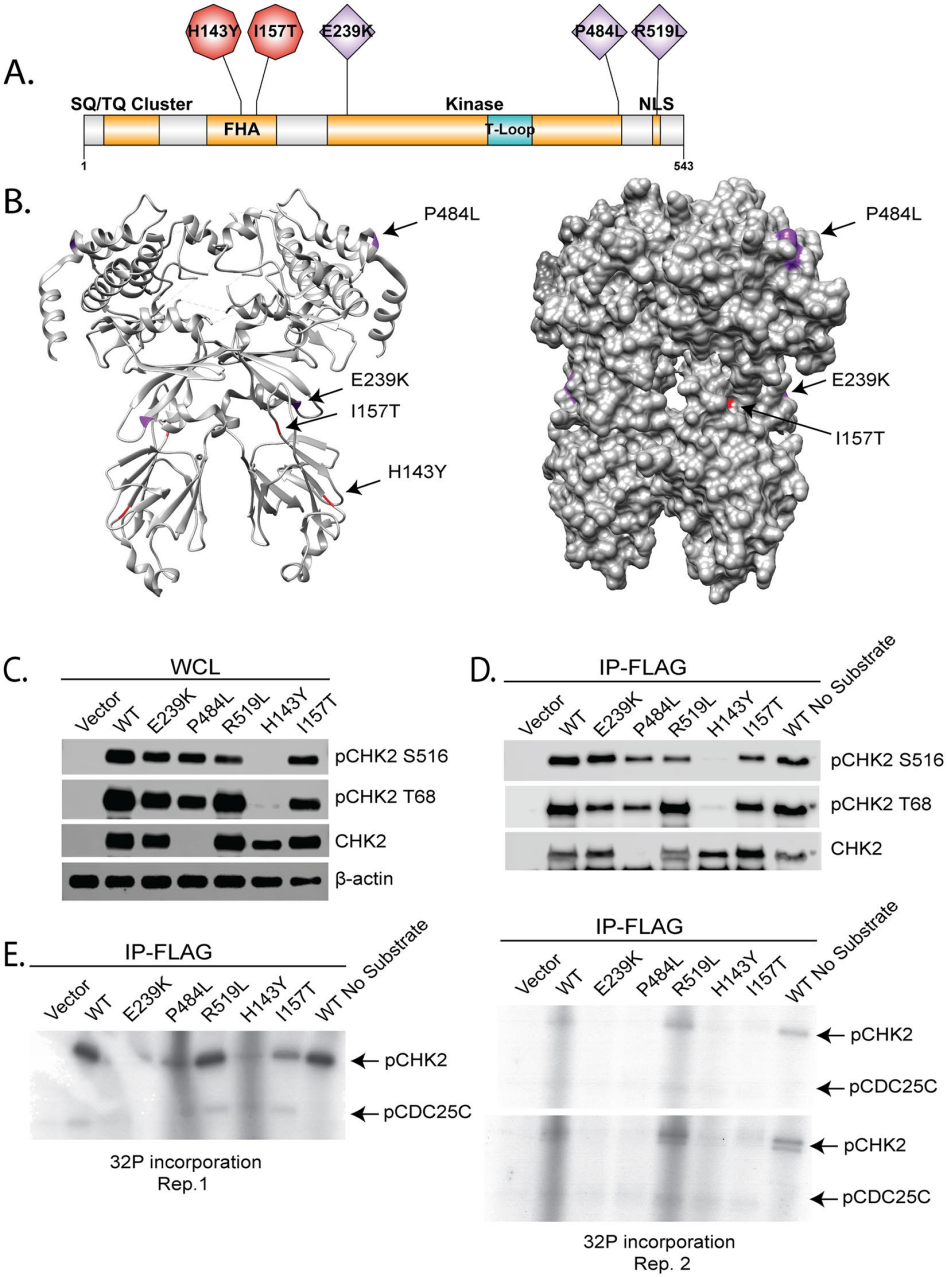
(**A**) Diagram human CHK2 protein domains and variants tested in kinase assays in the current study (purple) or known pathogenic variants (red). (**B**) Ribbon and Surface view of human CHK2 dimer (aa 89–501) with variants tested in this study (purple) and known pathogenic variants (red) (PDB ID: 3I6U). (**C**) Western blot analysis of CHK2 expression and activation in HEK293T expressing wild-type CHK2 or the indicated missense variants. Cells were exposed to 6 Gy IR and WCL (whole cell lysates) were extracted 1 h post-exposure. (**D**) Immunoprecipitated FLAG-CHK2 complexes from HEK293T lysates were immunoblotted for CHK2 and phospho-CHK2. E. *In vitro* kinase assays from HEK293T cell lysates. FLAG-CHK2 immunoprecipitates were incubated with a purified GST-CDC25C (aa 200–256) peptide. Both auto-phosphorylation of CHK2 and phosphorylation of CDC25C were used as a measure of CHK2 kinase activity.

Variant p.Pro484Leu, located at the end of the kinase domain showed dramatically decreased levels of expression when compared with the wild type (Fig. 3C,D). Levels of expression for p.Pro484Leu were lower than for both pathogenic variants p.His143Tyr and p.Ile157Thr, but differently from p.His143Tyr it still retains the ability to auto-phosphorylate at Serine 516 and to be phosphorylated by ATM at Threonine 68 (Fig. 3C,D). Variants p.Glu239Lys and p.Arg519Leu showed levels comparable to the wild type CHK2 (Fig. 3C,D).

In an *in vitro* kinase assay, p.Glu239Lys and p.Pro484Leu showed markedly decreased kinase activity comparable to the known pathogenic variant while variant p.Arg519Leu resulted in substrate phosphorylation levels similar to wild type CHK2 (Fig. 3E). In conclusion, functional analysis suggest that two CHEK2 variants found in our cohort (p.Glu239Lys and p.Pro484Leu) are likely to affect CHK2 activity *in vivo* and be associated with increased risk of breast cancer. While the evidence is less clear for p.Arg519Leu, this variant in the nuclear localization sequence and proximal to the Serine 516 site seems to auto-phosphorylate less efficiently (Fig. 3C,D).

### Non coding variants

To determine whether our patients harbored potentially functional variants in addition to protein alteration, we examined variants with the two highest functional score using two annotation tools: RegulomeDB (classes: 1a/f, 2a/b/c) and FunSeq (classes: 5, 4). Of the six variants most likely to be functional by both methods, two were near *CTNNA1*: one was considered to be in an ultra-sensitive region by FunSeq (11 probands), and the other was predicted to be motif breaking for *HDAC2* (3 probands). An *MSH6* intronic variant was predicted to be motif breaking for *ETS1*, *GABPA*, and *EGR1* (3 probands). A variant in the upstream promoter region of *STK11* was predicted to be motif breaking for *SP1* (1 proband). The promoter region of *ATM* harbored a variant predicted to be motif breaking for *BCL11A* and *BCL3* (1 proband). Finally, a *PALB2* promoter region variant is predicted to be motif breaking for *MAX*, *MYC*, and *USF1* (1 proband).

### Global and local ancestry in carriers of pathogenic variants

In the study cohort, the contribution of each ancestral population to the genome was 74.3% (SD 16.2), 14.9% (SD 12.2) and 10.8% (SD 7.4) for European, African and Native American ancestry, respectively (Supplementary Fig. 4,A). Carriers of a pathogenic variant were distributed throughout the spectrum of global ancestries. On the *CHEK2* locus, both carriers of the p.Ile157Thr pathogenic variant were homozygous for European ancestry (Supplementary Fig. 4,B). The *MUTYH* p.Gly396Asp pathogenic variant was also located in a European ancestral context (Supplementary Fig. 4,C). In contrast, the *RAD51B* p.Arg47* variant was located on a chromosome segment of African origin (Supplementary Fig. 4,D).

## Discussion

This study aimed at identifying inherited pathogenic variants in *BRCA*-negative breast cancer cases from Puerto Rico. Pathogenic variants were identified in *CHEK2*, *MUTYH*, and *RAD51B* in four cancer patients, which represented 8.3% of the cohort. In addition, ~30% of the VUS identified were found in NCCN actionable genes. Our study reveals an unmet clinical need of genetic testing that could benefit a significant proportion of at-risk women.

We identified four carriers with pathogenic or likely pathogenic *CHEK2* variants (p.Ile157Thr in two unrelated individuals, p.Glu239Lys and p.Pro484Leu), which represents over 8% of our cohort. In ClinVar, the p.Ile157Thr variant (VCV000005591.3) currently has conflicting interpretations with most submissions representing Likely pathogenic (8) or Pathogenic (17/20). The Ile157 residue is central in the FHA-kinase domain interface and functionally analyses of this variant by several independent groups indicates that the substitution generates a partially defective protein^36–40^. The p.Ile157Thr variant may confer a modest increase in unselected and familial BC risk (OR ~ 1.5) and a high risk (OR > 4) for lobular breast cancer, although risk estimates are not available for Puerto Rican women^41^.

Two missense VUS (p.Glu239Lys and p.Pro484Leu), located in the N- and C-terminal lobes of the kinase domain^36,38^, respectively, were shown to have marked decreases in kinase activity, and may constitute pathogenic variants. Although not central to its stabilization, the p.Glu239Lys is also located at the FHA-Kinase dimerization interface^36^. A change from a negatively to a positively charged amino acid might have an impact on CHK2 dimerization. Pro484 faces away from the dimerization axes but replacing proline may be reflected in a less rigid positioning of the alpha helix adjacent to the C-terminal end of the kinase domain^36^. Of note, although p.Arg519Leu, located in the NLS region, only displayed mild effects, our assay did not evaluate its cellular distribution. It is currently a variant of uncertain significance.

Significant differences in the prevalence of *CHEK2* variants across ethnic groups have been observed in the literature^42^. The cancer spectrum in families of carriers of pathogenic or likely pathogenic *CHEK2* variants based on functional assays, was consistent with the literature including breast, early onset breast, prostate, and colorectal cancers. Remarkably, two out of four families reported multiple cases of second primary or bilateral breast cancers (PRI-177 III-2 and III-3; PRI-193 III-1 and IV-1), consistent with published meta-analysis reporting the association of *CHEK2* with second primary breast cancers^43^. Potentially pathogenic *CHEK2* variants were overrepresented in this cohort, suggesting that *CHEK2* variants may affect the Puerto Rico population disproportionately. We have previously described a founder effect for a recurrent *BRCA2* variant in Puerto Rico (p.Glu1308Ter)^13^, a phenomenon that is likely contributing to increased variant frequencies in this population.

One patient was heterozygous for a known pathogenic variant in *MUTYH*, with a family history of early onset colorectal cancer consistent with the reported cancer risk for this gene. *MUTYH*-associated polyposis is inherited as an autosomal recessive trait^44^, with bi-allelic carriers demonstrating up to 28-fold increase in colorectal cancer risk^45^. Mono-allelic carriers have modest increases in risk of colorectal cancer, which was shown to differ in magnitude based on the variant^46,47^ and family history of colorectal cancer^47^. There is also evidence supporting a role of *MUTYH* in extra colonic tumor risk^48^, including emerging associations with the risk of BC, as two missense variants in *MUTYH* were shown to confer a significant increase in breast cancer risk in Sephardic Jews, a population in which the *MUTYH* mutation prevalence was high^49^. As of now, the data supporting the association between monoallelic *MUTYH* pathogenic variants and BC risk remains sporadic and additional evidence will be required before management guidelines are modified for this gene.

A *RAD51B* truncating variant p.Arg47* was observed in a family characterized by early onset breast cancer and colorectal cancer. Although a segregation analysis would be required to assess the carrier status of relatives with colorectal cancer, to our knowledge, there has been no report of the association of *RAD51B* variant with the familial risk of colorectal cancer. In a French study, *RAD51B* likely deleterious variants were observed in both breast-ovarian and breast-only cancer families^50^. GWAS have consistently identified the 14q24.1 genomic interval locus as a breast cancer susceptibility locus, which can be broken down into 2 signals mapping within or close to *RAD51B*. The first has been shown to be associated with triple negative phenotype and mammographic density^51^, and the second with male breast cancer risk^52^. When combined with GWAS evidence, our results suggest that *RAD51B* may be a candidate that warrants reconsideration for inclusion in breast cancer susceptibility gene panels. The local ancestry at the *RAD51B* locus in the carrier of p.Arg47* was predicted to be of African origin, which highlights the complexity of Hispanic populations in studying genetic basis to disease as pathogenic factor may originate from any of the ancestral populations that make up their genetic backgrounds. The identification of pathogenic variants in genes not commonly included in hereditary cancer testing is a reminder that negative results, especially in the context of strong family history, should be interpreted with care.

In the current study, over 46% of the study participants were found to carry a non-synonymous coding VUS. In the *BRCA1*/*2* genes, previous studies have reported higher rates of novel mutations or VUS in historically understudied populations^53–55^. In the clinic, VUS are associated with additional challenges. In U.S community-based practices, clinicians reported less confidence in interpreting and counseling multigene panel testing for cancer risk assessment when compared to single-gene testing^56^. In a survey among members of the National Society of Genetic Counselors, only 63.2% felt their patients understood the meaning of a VUS result^57^. For counselees, VUS results are less understood^58^ and have been shown to be associated with psychological distress^59^. The BROCA panel was developed to provide comprehensive testing for hereditary cancers, and includes several genes that have not been associated with BC risk. While standardized panel simplifies genetic testing, it also results in a higher probability of identifying VUS, which remain challenging in the clinic.

In 42.9% of the cohort, no potential causative variant was identified. Familial aggregation and twin studies have shown that even in non-hereditary breast cancer cases, a significant proportion of risk is inherited^60,61^. To date, genome-wide association studies (GWAS) have identified over 100 loci associated with breast cancer risk^62^, accounting for up to 14% of the familial risk attributed to common variants^63^. Although the risk associated with individual GWAS loci is not elevated enough to inform clinical decisions, polygenic risk scores were proposed as risk stratification tools in population screening programs and targeted prevention^64–67^. It is possible that high-risk families for which no potential causative variant is identified share an excess of low penetrance variants.

Although this is the first study addressing specifically hereditary cancer risks in an entirely Puerto Rican population, the sample size was limited. In addition, because the inclusion criteria required a *BRCA1/2* negative clinical test result, patients of higher socio-economic status with better access to health care are likely to be overrepresented in this cohort. While the clinic where recruitment took place serves patients from all over the island, its location in the San Juan metropolitan area probably favored the recruitment of patients from the metropolitan area. The European contribution to the global genetic ancestry of the cohort was estimated at 74.3%, which is higher than the previously reported average of 63.7% for the Island in a census based population^68^. European ancestry in Puerto Rico has been associated with higher socio-economic status^68^. Consequently, having access to *BRCA* genetic testing, which was an inclusion criterion in this study, is likely to be correlated with a higher socio-economic status and might explain this enrichment in European ancestry.

Our understanding of the genetic factors driving disease risk is biased towards populations of European descent^69^. This is especially concerning, as it has been documented that rarer variants are more likely to show geographic clustering. In this study, we provide the first description of the pathogenic variants underlying BC genetic risk in Puerto Rican Hispanic women who tested negative for a *BRCA* mutation. Over 12% of the study participants had a known pathogenic variant or a variant that was predicted pathogenic by functional assay. For a large proportion of the genes included in hereditary cancer gene panels, clinical guidelines for management of risk have yet to be established pending the availability of accurate estimates of their respective cancer spectrum and the magnitude of associated risk.

## Supplementary information

Supplementary tables and figures
