# Supplementary material for: Germline variants in cancer genes in high-risk non-BRCA patients from Puerto Rico
Source: Sci Rep. 2019 Nov 28;9:17769. doi: 10.1038/s41598-019-54170-6 (PMC6882826; doi:10.1038/s41598-019-54170-6)
Supplement: Supplementary file 1 — Supplementary tables and figures [file 41598_2019_54170_MOESM1_ESM.pdf]

Supplementary materials

Germline variants in cancer genes in high-risk non-*BRCA* patients from Puerto Rico

Julie Dutil, Jamie K Teer, Volha Golubeva, Sean Yoder, Wei Lue Tong, Nelly Arroyo, Rachid Karam, Miguel Echenique, Jaime L. Matta, Alvaro N. Monteiro

Supplementary Table 1. Demographic and clinical characteristics of the study cohort.

		n (%)
Demographic variables		
Age	<40yrs	8 (16.7)
	40-49	12 (25.0)
	50-59	13 (27.1)
	60-69	9 (18.8)
	≥70	6 (12.5)
BMI	<25	20 (41.7)
	25-29	21 (43.8)
	≥30	7 (14.6)
Civil status	Single	10 (20.8)
	Married	30 (62.5)
	Divorced	7 (14.6)
	Widow	1 (2.1)
Education level	Up to high school	3 (6.3)
	Associate	7 (14.6)
	Bachelor	38 (79.2)
Hormonal & pregnancy history		
Age at menarche	≥13	15 (32.6)
	<13	31 (67.4)
Oophorectomy	Unilateral	4 (8.7)
	Bilateral	4 (8.7)
Oral contraceptive	Never used	17 (63.8)
	Ever used	30 (36.2)
Pregnancy	Ever been pregnant	40 (85.1)
	Never been pregnant	7 (14.9)
Number of children	Nulliparous	10 (21.3)
	1-2	25 (53.2)
	≥3	12 (25.5)
Age at first child	<20	3 (7.9)
	20-29	27 (71.1)
	≥30	8 (21.1)
Breast feeding	Never	11 (32.4)
	<12 months	20 (58.8)
	≥12 months	3 (8.8)
Menopause[1]	Premenopausal	19 (42.2)
	Peri-/post-menopausal	26 (57.8)
Age menopause	<40	3 (13.6)
	40-49	9 (40.9)
	≥50	10 (45.5)
Estrogen replacement therapy	Ever used	7 (15.6)
	Never used	38 (84.4)

Continued on next page.

Supplementary Table 1. Continued.

		n (%)
Clinical parameters & pathology		
Age of diagnosis	<40yrs	11 (22.9)
	40-49	16 (33.3)
	50-59	7 (14.6)
	60-69	13 (27.1)
	≥70	1 (2.1)
Site/type	Ductal carcinoma, *in situ*	9 (20.5)
	Ductal carcinoma, invasive	29 (65.9)
	Lobular carcinoma, invasive	6 (13.6)
Grade (invasive tumors)	I	8 (25.8)
	II	16 (51.6)
	III	17 (22.6)
Size (cm)	<2	20 (76.9)
	≥2	6 (23.1)
ER	negative	4 (11.8)
	positive	30 (88.2)
PR	negative	13 (38.2)
	positive	21 (61.8)
HER2	negative	25 (86.2)
	positive	4 (13.8)
IHC subtype	triple negative	3 (10.7)
	all others	25 (89.3)
Lymph nodes	positive	6 (22.2)
	negative	21 (77.8)

[1]Peri-/post-menopause includes natural and induced menopause. *The total number of subjects varies across variables because of missing values (n missing was age at menarche 2, pregnancy 1, age at first child 10, number of children 1, breast feeding duration 14, menopause 3, age at menopause 7, estrogen replacement therapy 3, tumor site/type 4, grade 8, size 22, ER 14, PR 14, HER2 19, IHC subtype subtype 20). BMI body mass index, cm centimeters ER estrogen receptor, IHC immunohistochemistry, PR progesterone receptor, HER2 human epidermal growth factor receptor 2.

Supplementary Table 2. Frequencies for non-synonymous L/Pathogenic variants and VUS reported in gnomAD.

				gnomAD frequencies[1]		
Gene	Chr:position	HCVS (c)	HCVS (p)	African	European	Latino
APC	5:112177928	c.6637A>G	p.Met2213Val	4.95E-04	0.00E+00	2.83E-05
ATM	11:108098389	c.38G>A	p.Arg13His	0.00E+00	1.76E-05	2.89E-05
	11:108121787	c.1595G>A	p.Cys532Tyr	1.23E-04	2.25E-04	8.19E-04
	11:108160480	c.4388T>G	p.Phe1463Cys	2.81E-04	5.27E-04	1.10E-03
	11:108196144	c.6680G>A	p.Arg2227His	0.00E+00	8.81E-06	0.00E+00
	11:108202716	c.7740A>C	p.Arg2580Ser	0.00E+00	4.66E-05	2.26E-04
	11:108225561	c.8810T>C	p.Val2937Ala	0.00E+00	0.00E+00	1.18E-03
BLM	15:91304122	c.1519G>A	p.Glu507Lys	0.00E+00	0.00E+00	8.68E-05
	15:91326057	c.2561G>A	p.Ser854Asn	0.00E+00	0.00E+00	8.70E-05
	15:91358475	c.4220G>A	p.Arg1407Lys	0.00E+00	7.74E-06	2.54E-04
BMPR1A	10:88683138	c.1348G>A	p.Val450Met	0.00E+00	1.76E-05	1.45E-04
BRCA2	13:32913588	c.5096A>G	p.Asp1699Gly	0.00E+00	9.34E-06	7.02E-05
	13:32936785	c.7931A>G	p.Asn2644Ser	0.00E+00	8.80E-06	0.00E+00
BRCC3	X:154305469	c.220A>G	Ile74Val	5.47E-05	1.23E-03	2.15E-04
CDH1	16:68853185	c.1568A>G	p.Tyr523Cys	0.00E+00	7.74E-06	1.13E-04
CHEK1	11:125503187	c.554A>G	p.His185Arg	0.00E+00	2.36E-05	0.00E+00
CHEK2	22:29121087	c.599T>C	p.Ile157Thr	8.01E-05	5.31E-03	5.64E-05
	22:29107974	c.715G>A	p.Glu239Lys	0.00E+00	5.42E-05	2.54E-04
	22:29090030	c.1451C>T	p.Pro484Leu	8.53E-05	8.05E-05	2.56E-04
	22:29083961	c.1556G>T	p.Arg519Leu	4.31E-05	1.70E-04	4.55E-04
GALNT12	9:101589171	c.669C>G	p.Leu227Val	0.00E+00	0.00E+00	2.83E-03
GEN1	2:17954003	c.905G>A	p.Arg302His	9.63E-04	4.57E-03	1.42E-03
	2:17963043	c.2564G>A	p.Arg855Lys	0.00E+00	2.66E-05	0.00E+00
MRE11A	11:94219093	c.311G>C	p.Ser104Thr	0.00E+00	0.00E+00	5.65E-05
MSH6	2:48027040	c.1921_1923delGAA	p.Glu641del	NA	NA	NA
MUTYH	1:45800158	c.62C>T	p.Ala21Val	NA	NA	NA
	1:45797228	c.1187G>A	p.Gly396Asp	1.02E-03	4.89E-03	3.08E-03
NBN	8:90982716	c.772G>C	p.Glu258Gln	NA	NA	NA
PIK3CA	3:178916753	c.140A>G	p.His47Arg	4.55E-04	0.00E+00	1.71E-04
PMS2	7:6027134	c.1262G>A	p.Arg421Gln	0.00E+00	0.00E+00	2.89E-05
POLD1	19:50902657	c.232C>T	p.Arg78Cys	2.40E-04	1.55E-05	0.00E+00
POLE	12:133245025	c.2090C>G	p.Pro697Arg	0.00E+00	0.00E+00	1.46E-04
PPM1D	17:58678214	c.439C>G	p.Leu147Val	4.20E-05	2.38E-04	9.63E-04
PRSS1	7:142459867	c.443C>T	p.Ala148Val	2.56E-03	2.24E-04	1.99E-04
RAD51B	14:68292235	c.139C>T	p.Arg47*	4.01E-05	9.29E-05	5.65E-05
	14:68353784	c.619G>T	p.Val207Leu	0.00E+00	9.03E-06	0.00E+00
SDHB	1:17349152	c.716C>G	p.Ser239Cys	0.00E+00	1.23E-04	0.00E+00
	1:17355201	c.317A>G	p.Asn106Ser	1.15E-04	0.00E+00	0.00E+00

[1] GnomAD genome aggregation database (https://gnomad.broadinstitute.org/, accessed August 10, 2019), European refers to non-Finnish European.

Supplementary Table 3. *In silico* functional prediction models for non-synonymous L/Pathogenic variants and VUS.

Gene	Chr:position	HCVS (c)	HCVS (p)	fathmm (score)	fathmm (classification)	MutationAssessor (score)	MutationAssessor (predicted impact)	PolyPhen-2 (probability)	PolyPhen-2 (classification)	SIFT (score)	SIFT (classification)
APC	5:11217792	c.6637A>G	p.Met2213Val	-2.39	Damaging	0	Neutral	0	Benign	0.316	Tolerated
ATM	11:1080983	c.38G>A	p.Arg13His	0.17	Tolerated	1.765	Low	0.022	Benign	0.195	Tolerated
	11:1081217	c.1595G>A	p.Cys532Tyr	-0.96	Tolerated	2.71	Medium	1	Probably damaging	0.001	Damaging
	11:1081604	c.4388T>G	p.Phe1463Cy	-0.33	Tolerated	2.51	Medium	1	Probably damaging	0.001	Damaging
	11:1081961	c.6680G>A	p.Arg2227His	-2.3	Damaging	2.67	Medium	1	Probably damaging	0	Damaging
	11:1082027	c.7740A>C	p.Arg2580Ser	-1.2	Tolerated	2.71	Medium	0.001	Benign	0.132	Tolerated
	11:1082255	c.8810T>C	p.Val2937Ala	-1.37	Tolerated	1.93	Low	1	Probably damaging	0.032	Damaging
BLM	15:9130412	c.1519G>A	p.Glu507Lys	0.78	Tolerated	2.135	Medium	0.792	Possibly damaging	3.54	Damaging
	15:9132605	c.2561G>A	p.Ser854Asn	0.89	Tolerated	1.385	Low	0.026	Benign	2.82	Tolerated
	15:9135847	c.4220G>A	p.Arg1407Lys	0.1	Tolerated	2.565	Medium	0.994	Probably damaging	3.22	Damaging
BMPR1A	10:8868313	c.1348G>A	p.Val450Met	-3.22	Damaging	1.605	Low	0.237	Benign	0.099	Tolerated
BRCA2	13:3291358	c.5096A>G	p.Asp1699Gl	5.68	Tolerated	1.935	Low	0.006	Benign	0.21	Tolerated
	13:3293678	c.7931A>G	p.Asn2644Se	-1.96	Damaging	0.705	Neutral	0.034	Benign	0.192	Tolerated
BRCC3	X:15430546	c.220A>G	Ile74Val	0.43	Tolerated	1.15	Low	0.278	Benign	0.05	Damaging
CDH1	16:6885318	c.1568A>G	p.Tyr523Cys	0.03	Tolerated	4.2	High	1	Probably damaging	0	Damaging
CHEK1	11:1255031	c.554A>G	p.His185Arg	1.84	Tolerated	0.4	Neutral	0	Benign	0.535	Tolerated
CHEK2	22:2912108	c.599T>C	p.Ile157Thr	0.995	Probably dam.	-0.14	Tolerated	0.365	neutral	0.173	Tolerated
	22:2910797	c.715G>A	p.Glu239Lys	0.61	Possibly dam.	-0.22	Tolerated	0.895	low	0.011	Damaging
	22:2909003	c.1451C>T	p.Pro484Leu	0.514	Possibly dam.	-2.05	Damaging	0.83	low	0.062	Tolerated
	22:2908396	c.1556G>T	p.Arg519Leu	0.124	Tolerated	0.01	Tolerated	0.69	neutral	0.712	Tolerated
GALNT12	9:10158917	c.669C>G	p.Leu227Val	-0.27	Tolerated	3.28	Medium	0.999	Probably damaging	0.001	Damaging
GEN1	2:17954003	c.905G>A	p.Arg302His	0.88	Tolerated	1.78	Low	0.971	Probably damaging	0.025	Damaging
	2:17963043	c.2564G>A	p.Arg855Lys	1.91	Tolerated	0.695	Neutral	0	Benign	0.585	Tolerated
MRE11A	11:9421909	c.311G>C	p.Ser104Thr	-0.92	Tolerated	0.92	Low	0.062	Benign	0.224	Tolerated
MSH6	2:48027040	c.1921_1923delGA	p.Glu641del	NA	NA	NA	NA	NA	NA	NA	NA
MUTYH	1:45800158	c.62C>T	p.Ala21Val	3.29	Tolerated	1.625	low	0.335	Benign	0.006	Damaging
	1:45797228	c.1187G>A	p.Gly396Asp	-3.78	Damaging	4.09	High	1	Probably damaging	0	Damaging
NBN	8:90982716	c.772G>C	p.Glu258Gln	0.28	Tolerated	2.48	Medium	0.998	Probably damaging	0.106	Tolerated
PIK3CA	3:17891675	c.140A>G	p.His47Arg	-0.63	Tolerated	0.695	Neutral	0.122	Benign	0.452	Tolerated
PMS2	7:6027134	c.1262G>A	p.Arg421Gln	1.09	Tolerated	2.095	Medium	0.998	Probably damaging	0.098	Tolerated
POLD1	19:5090265	c.232C>T	p.Arg78Cys	2.1	Tolerated	0.55	Neutral	0.998	Probably damaging	0.019	Damaging
POLE	12:1332450	c.2090C>G	p.Pro697Arg	2.35	Tolerated	2.65	Medium	0.544	Possibly damaging	0.052	Tolerated
PPM1D	17:5867821	c.439C>G	p.Leu147Val	2	Tolerated	0.74	Neutral	0.076	Benign	0.496	Tolerated
PRSS1	7:14245986	c.443C>T	p.Ala148Val	-2.48	Damaging	0.81	Low	0	Benign	0.188	Tolerated
RAD51B	14:6829223	c.139C>T	p.Arg47*	NA	NA	NA	NA	NA	NA	NA	NA
	14:6835378	c.619G>T	p.Val207Leu	-0.37	Tolerated	0.59	Neutral	0.058	Benign	0.698	Tolerated
SDHB	1:17349152	c.716C>G	p.Ser239Cys	-4.23	Damaging	3.885	High	1	Probably damaging	0.001	Damaging
	1:17355201	c.317A>G	p.Asn106Ser	-6	Damaging	2.82	Medium	0.078	Benign	0.03	Damaging

L/Pathogenic Likely pathogenic or pathogenic, VUS variants of unknown significance, dam. damaging.

Supplementary **Figure 1**

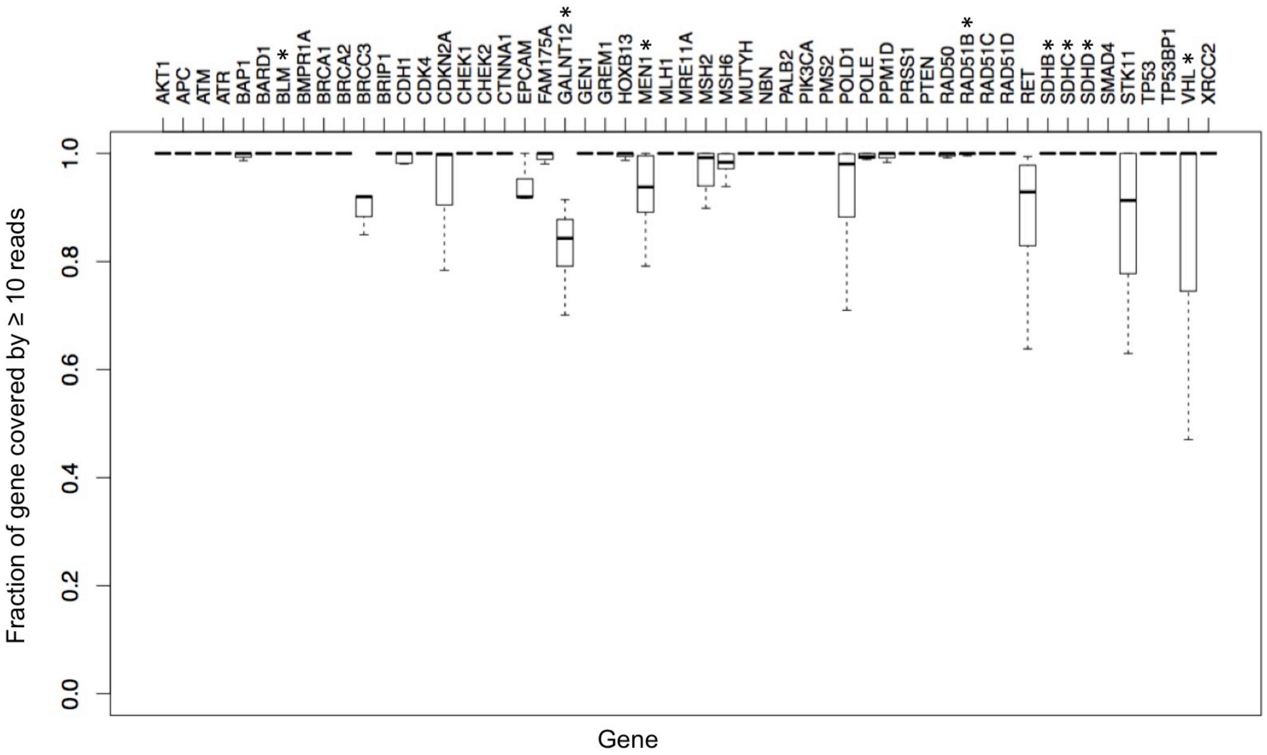

Legend to **supplementary Figure 1**. BROCA capture panel gene list and fraction of each gene covered by 10 or more reads. (*) Indicate genes for which only exons were targeted by capture. For other genes, capture probes include exons, introns and regulatory regions. Gene symbols are according to HGNC-approved nomenclature.

Supplementary **Figure 2**

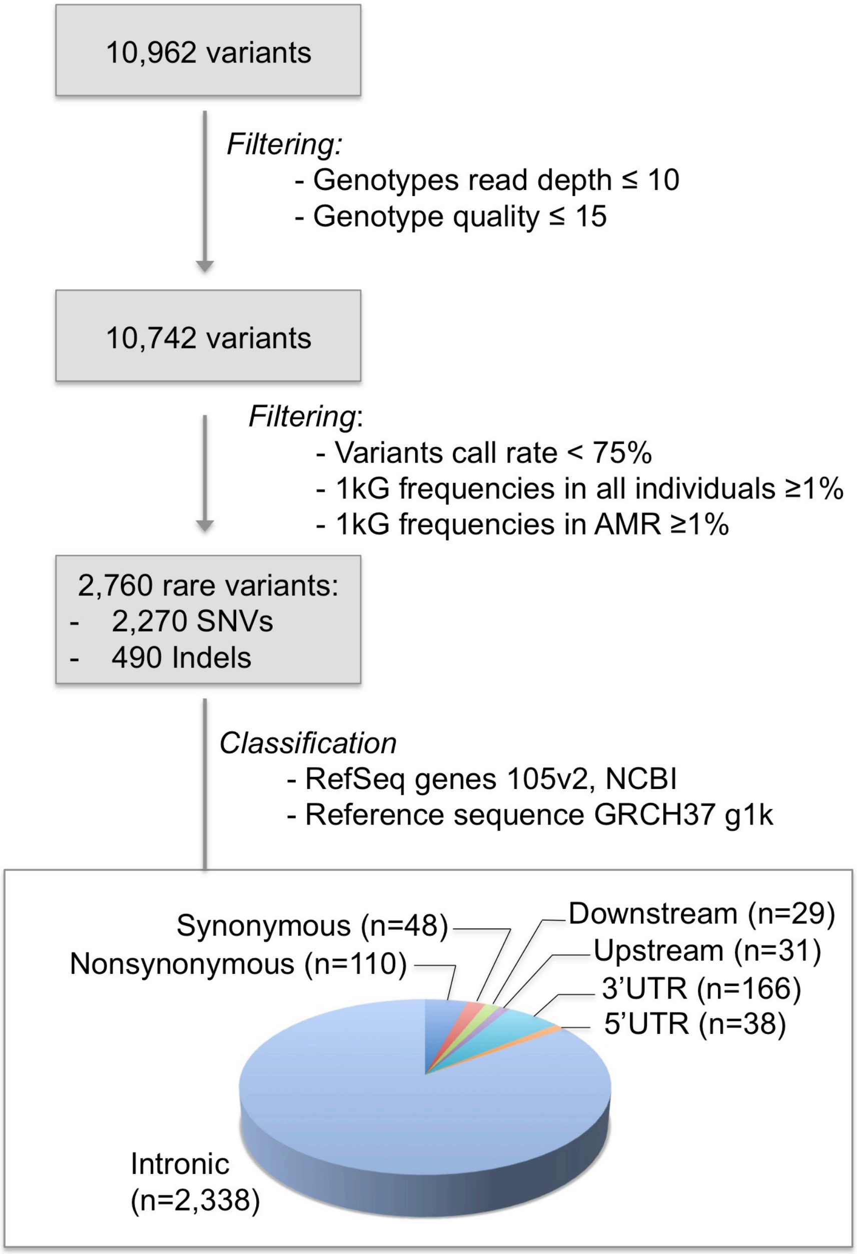

Legend to **supplementary Figure 2**. Variant filtering, classification and annotation. A total of 10,962 variants were identified in 52 individuals from 49 families. Genotypes were set to missing when read depth ≤ 10 and/or genotype quality ≤ 15. Variants with call rates of less than 75% were excluded from further analysis. After applying quality filters, there were 7,742 variants left. Using 1000 Genomes allelic frequency data (2013-05-02 sequence and alignment release, all individuals and AMR), variants present in the 1kG database and with frequency ≥ 0.01 were filtered out. There were 2,760 variants remaining, including 2,270 SNVs and 490 Indels. Those correspond to rare variants in the 1kG database or variants that have not been reported by 1kG. Variants were further classified according to their predicted effect on protein (RefSeq gene 105v2 NCBI, reference sequence GRCH37 g1k). There were a total of 158 coding variants (48 synonymous, 110 missense, including 2 truncating and 2 deletions). The remaining were upstream (n=31), downstream (n=29), 3'UTR (n=166), 5'UTR (n=38), intronic (2,338).

Supplementary **Figure 3**

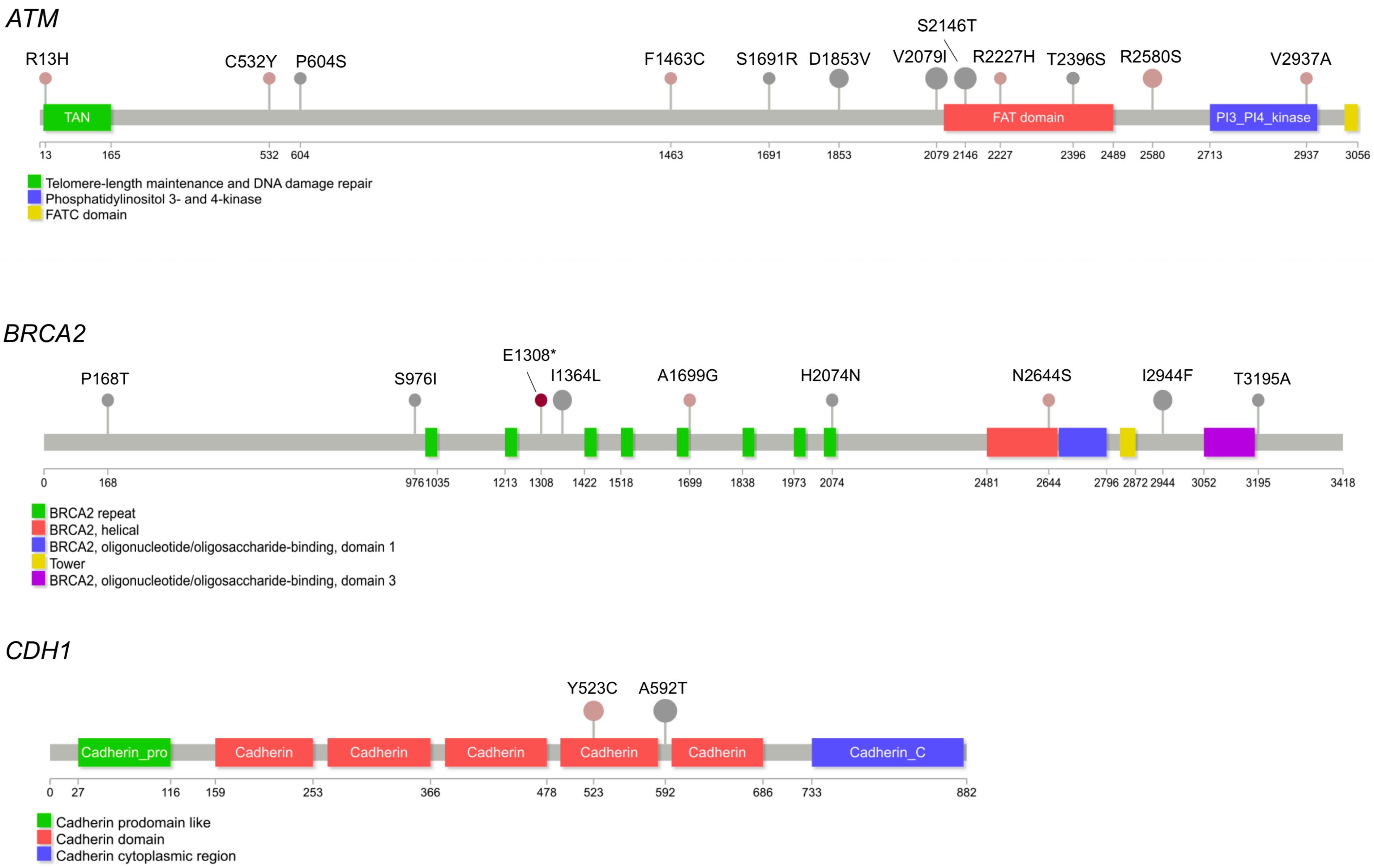

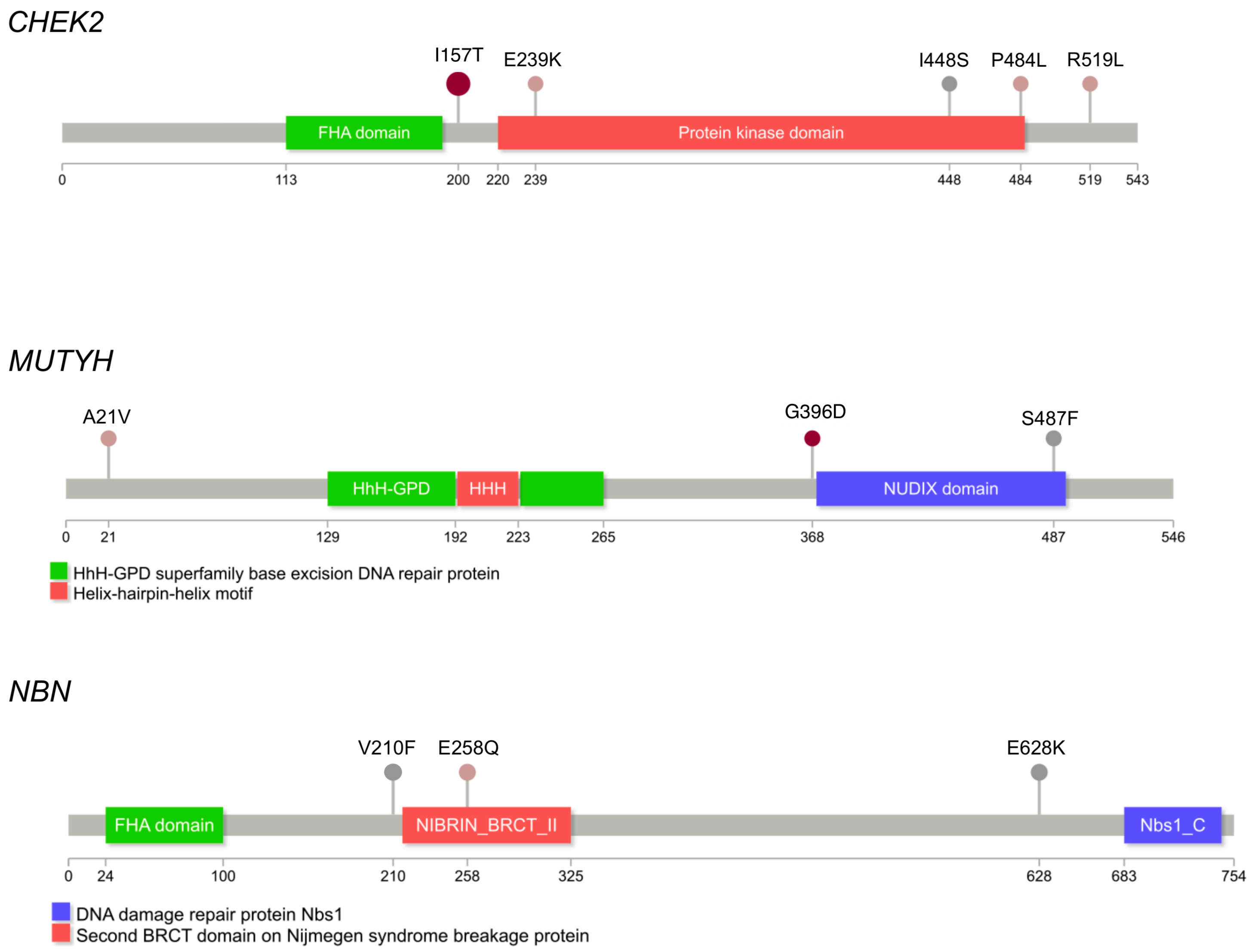

CHEK2
I157T
E239K
I448S
P484L
R519L
FHA domain
Protein kinase domain
0
113
200
220
239
448
484
519
543
MUTYH
A21V
G396D
S487F
HhH-GPD
HHH
NUDIX domain
0
21
129
192
223
265
368
487
546
HhH-GPD superfamily base excision DNA repair protein
Helix-hairpin-helix motif
NBN
V210F
E258Q
E628K
FHA domain
NIBRIN_BRCT_II
Nbs1_C
0
24
100
210
258
325
628
683
754
DNA damage repair protein Nbs1
Second BRCT domain on Nijmegen syndrome breakage protein

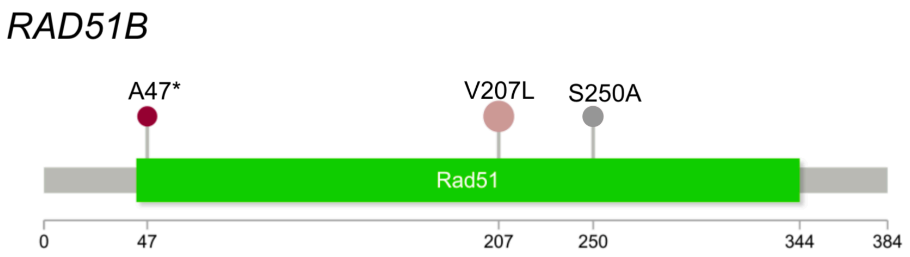

Legend to **supplementary Figure 3**. Coding variant distribution in genes harboring likely pathogenic/pathogenic (L/Pathogenic) variants and in breast cancer NCCN clinically actionable breast cancer genes in which variants of unknown significance (VUS) have been identified. Known L/Pathogenic variants, VUS and benign/likely benign variants are represented by dark red, pink and grey lollipops, respectively. The size of the lollipops is proportional to the number of observations of a given variant in unrelated individuals of the study population (range 1-4). Includes non-synonymous coding variants (stopgain, deletions and single amino acid changes). Genes for which only likely benign/benign variants have been identified or genes that are not classified as clinically actionable by NCCN are not included in that figure.

Supplementary **Figure 4**

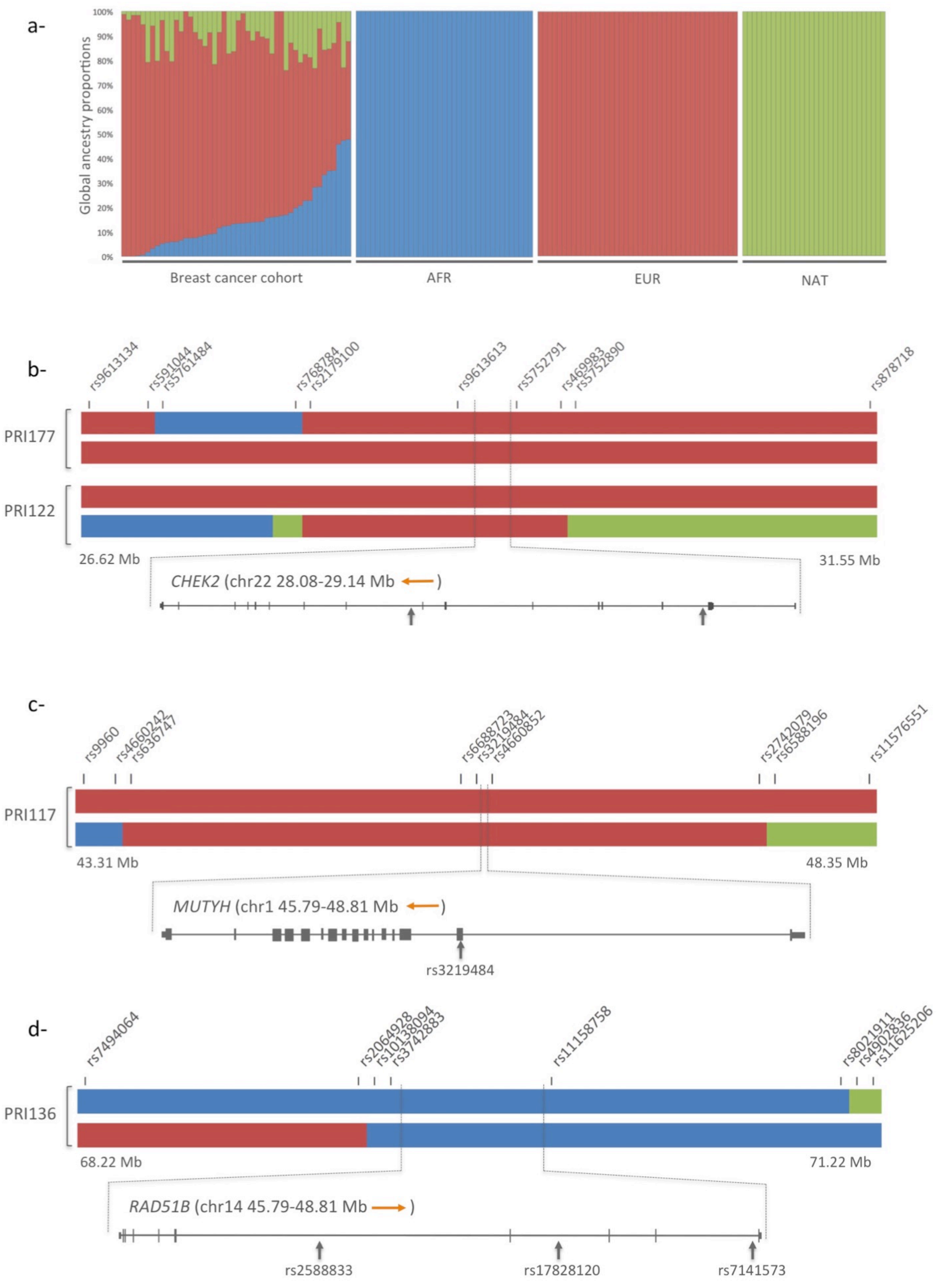

Legend to **supplementary Figure 4**. Global ancestry proportion (a) in study population in comparison to reference ancestral populations (African AFR, European EUR, and Native American NAT). Local ancestry estimates in known pathogenic variants carriers at the *CHEK2* (b), *MUTYH* (c), and *RAD51B* (d) loci.
